# Evaluation of the microleakage of class V composite restoration after cavity treatment with Erbium, CO_2_ lasers, Papain, and Bromelain enzymes

**DOI:** 10.1002/cre2.822

**Published:** 2023-11-30

**Authors:** Farahnaz Sharafeddin, Anahita Fadaei Tabrizi

**Affiliations:** ^1^ Department of Operative Dentistry, Biomaterials Research Center, School of Dentistry Shiraz University of Medical Sciences Shiraz Iran; ^2^ Biomaterials Research Center, School of Dentistry Shiraz University of Medical Science Shiraz Iran

**Keywords:** Bromelain, CO_2_ laser, Erbium lase, Papain

## Abstract

**Objectves:**

Different surface preparation and treatment methods may have dissimilar effects on the microleakage of composite resin. This study was conducted to determine the deproteinizing effect of 10% bromelain enzyme, 10% papain enzyme, CO_2_, and erbium‐YAG laser in regard to decrease in the microleakage of composite restorations.

**Materials and Methods:**

Thirty teeth were selected and 60 class V cavities were prepared on the lingual and buccal sides. They were divided into six groups (*n* = 10): Group 1, phosphoric acid gel; Group 2, bromelain enzyme 10%; Group 3, papain enzyme 10%; Group 4, mixed papain and bromelain enzymes 10%; Group 5, CO_2_ laser; and Group 6, erbium‐YAG laser. They were stored in basic fuchsine and dye penetration was evaluated. Kruskal–Wallis and Mann–Whitney tests were used for statistical analysis, *p* < 0.05

**Results:**

In both occlusal and gingival margins, comparison of microleakage between groups 1, 2, 3, 4, and 5 showed no significant differences (*p* = 1) and group 6 had a significant difference with other groups (*p* ˂ 0.001).

**Conclusions:**

Microleakage of composite resin in the dentin surface was not affected significantly using either bromelain or papain 10% enzymes or erbium laser. However, CO_2_ laser had a negative effect on the enamel and dentin margins and increased the microleakage. Erbium laser showed a better effect than enzymes on microleakage.

## 1 INTRODUCTION

Microleakage is the invisible penetration of fluids, bacteria, molecules, or ions between the cavity and the restoration that can cause tooth discoloration, hypersensitivity, recurrent caries, pulpal problems, and induced restoration failure (Sharafeddin et al., 2019). To reduce the effect of the tooth organic matrix on the adhesion of composites to the dentin substrate, various substances with deproteinizing characteristic were suggested (Sharafeddin & Safari, 2019). In recent years, the effects of deproteinization with bromelain and papain enzymes on microleakage have been studied. Papain and bromelain enzymes on the basis of their proteolytic action were introduced. Bromelain is a proteolytic enzyme extracted from the fruit or stem of pineapple (Sharafeddin & Haghbin, 2019). Protease enzymes can catalyze the hydrolysis of proteins to give amino acids (Dayem & Tameesh, 2013). Papain is obtained from the latex of *Carica papaya*. It is also a proteolytic cysteine enzyme with anti‐inflammatory and antibacterial properties and is used for removing debris from dentine safely without any adverse effect on the dentin because of the specificity of the enzyme (Lopez et al., 2007; Sharafeddin & Safari 2019).

Lately, new technologies for preparing enamel and dentin, such as laser irradiation, have been suggested. There has been increasing concern about the use of lasers for preparing the dentin and enamel surfaces; this technique is an alternative to phosphoric acid‐etching methods. Since the time of approval by the U.S. Federal Drug Administration for the usage of Er:YAG lasers for caries elimination, cavity preparation, and treatment of tooth surface, there have been fewer studies on the use of this technique in comparison to enzyme solution dental surface treatment in order to microleakage reduction in composite resin restorations (Basir et al., 2016; Donmez et al., 2013).

Low energy Er:YAG laser modified the dentin surface and caused a surface free from the derbies with open dentinal tubules. It was reported that the heat created by the Er:YAG laser can eliminate free radicals and modify the dentin to provide a more suitable surface for the bonding process (Basir et al., 2016).

CO_2_ laser concentrates high levels of energy in a small area. Energy is converted to heat and results in burning, melting, or vaporizing of the respective area in dentin (Rezaei et al., 2019). One study has shown that a laser could develop adhesion and minimize gap formation and microleakage (Otero et al., 2021). Thus, the present study aimed to evaluate the microleakage of cavities treated by Er:YAG laser and CO_2_ laser irradiation and bromelain and papain 10% enzyme in composite resin restorations and compare the microleakage scores with acid etched cavities in human molar teeth.

## 2 MATERIALS AND METHODS

**Figure 1:**
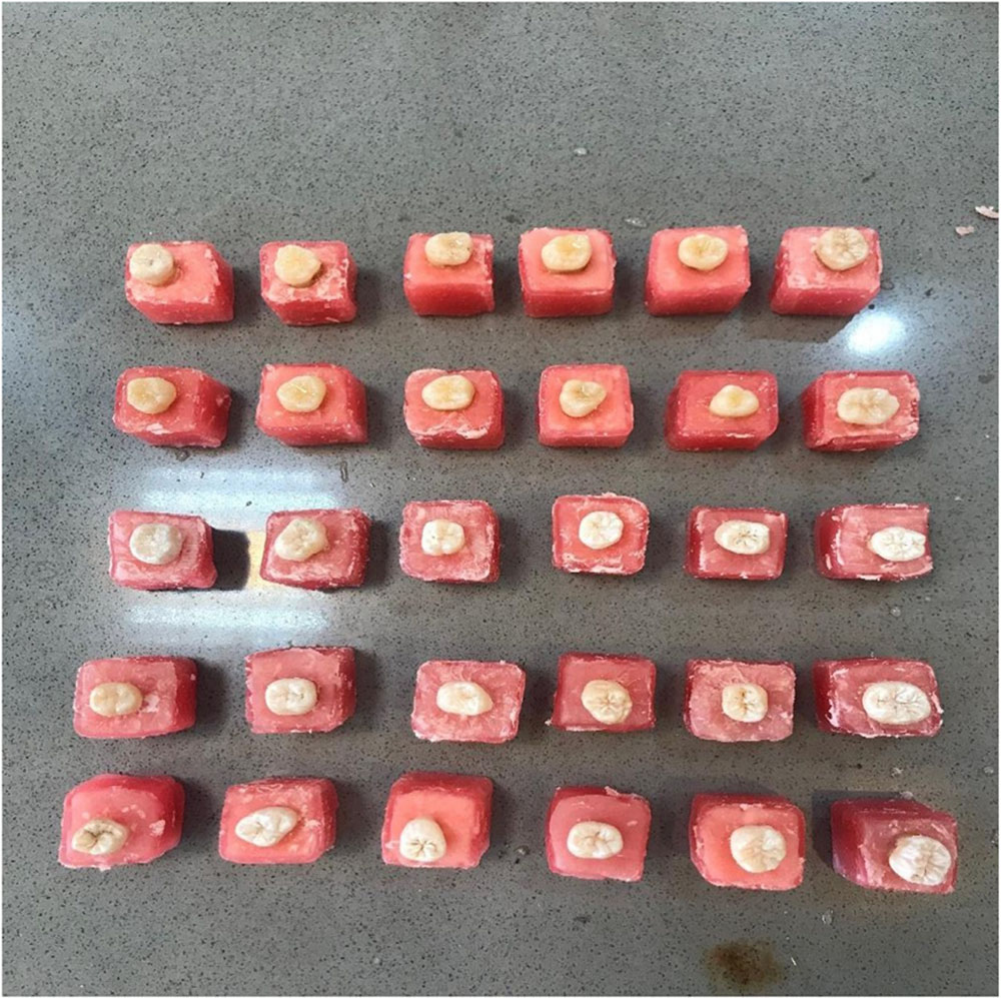
Experimental groups.

**Figure 2:**
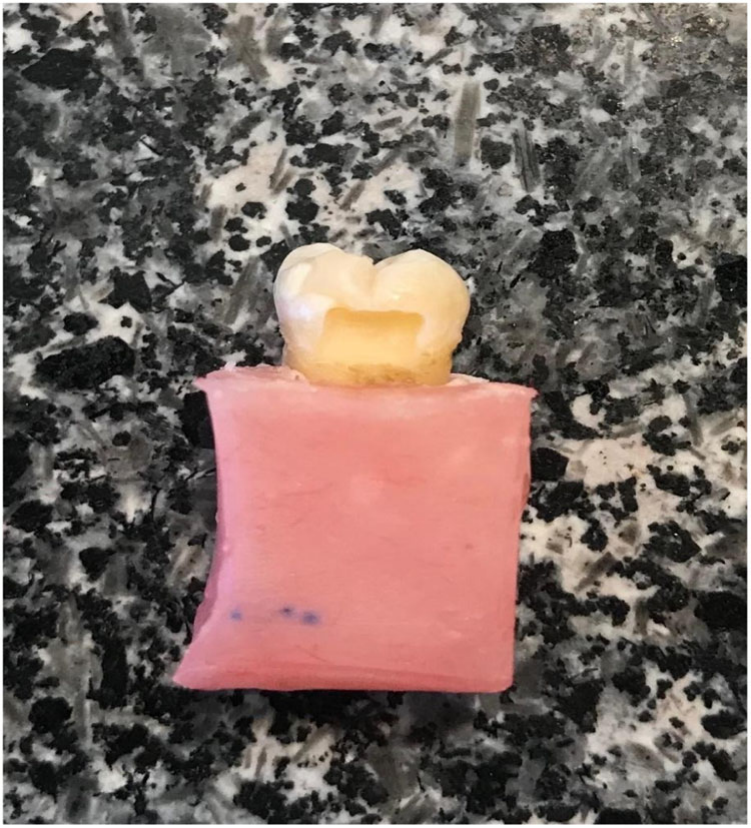
The class V cavity.

In this experimental study with ethical code IR.SUMS.DENTAL.REC.1398.037, 30 extracted sound mandibular molars were cleaned with a periodontal instrument and disinfected in 0.1% thymol solution. Then cleaned teeth were mounted in the acryl cubes from 3 mm under the cementoenamel junction (CEJ) (Figure 1). Fissure burs (Diamond fissure 330; Tizkavan) were used to prepare 60 class V cavities with gingival margins extending 1 mm under CEJ (5 mm in length, 3 mm in width, and 1.5 mm in depth) on the lingual and buccal surface of each tooth (Figure 2).

It was calculated based on a previous study (Salama et al., 2015). The estimated effect size between the study groups according to 10 samples for each group (*n* = 10) was determined to detect effect size (*f*) = 0.656 with type II error (*β*) = 0.20 at 80% level of power and significant level of 0.05.

The 10% bromelain and 10% papain enzyme solutions were prepared by mixing the enzyme powder (6 g) with distilled water (60 mL) in the biochemistry lab of shiraz University. The teeth were randomly assigned into six equal groups of 10 cavities (*N* = 5); group 1: the cavities were etched for 15 s with 37% phosphoric acid gel (DenFil) by a disposable microbrush in all parts of the cavity, group 2: the bromelain 10% solution (Biozyme) was applied into the cavities by a disposable microbrush for 1 min, group(2) 3: the papain 10% solution (Organica) was applied into the cavities by a disposable microbrush for 1 min, group 4: the mixed bromelain and papain 10% solution was applied into the cavities by a disposable microbrush for 1 min. All the prepared specimens were washed with distilled water (20 s) and air‐dried gently. Group 5: Er:YAG laser (erbium‐doped yttrium aluminum garnet laser; Profile) with a setting of 10 Hz, 50 mJ/cm^2^, 1.5 W, 300 μs pulse width and distance of 20 mm with a circular tip was used on the dentin surface, and group 6: CO_2_ laser (US‐20D; Deka Dental Lasers) with a setting of 10 Hz, 80 mJ/cm^2^, 1.5 W and distance of 20 mm with a circular tip was used on the surface of dentin for 30 s (materials and manufactures presented in Table 1).

**Table 1:** Manufactures and composition of materials.

Materials	Composition or parameters	Lot number and manufacture
NanohybrFiltek™Z350 XT A2 shade	Resin Matrix: Bis‐GMA, UDMA, TEGDMA, Filler content: 78.5 wt% (59.5 vol%) Silica, zirconia, aggregated zirconia/silica	NA92284 (3M ESPE)
Adper single bond2	HEMA, bis‐GMA, ethyl alcohol, silane‐treated silica, glycerol 1,3‐dimethacrylate, copolymer of acrylic and itaconic acids, diurethane dimethacrylate, water, 10% by weight of silica nanoparticles	N662648 (3M ESPE)
Er:YAG laser	Power: 1.5 W Energy: 50 mJ/cm^2^ pulse repetition: 10 Hz duration: 30 s	Profile
CO_2_ laser	Power: 1.5 W Energy: 80 mJ/cm^2^ pulse repetition: 10 Hz Duration: 30 s	US‐20D, Deka Dental Laser system

**Figure 3:**
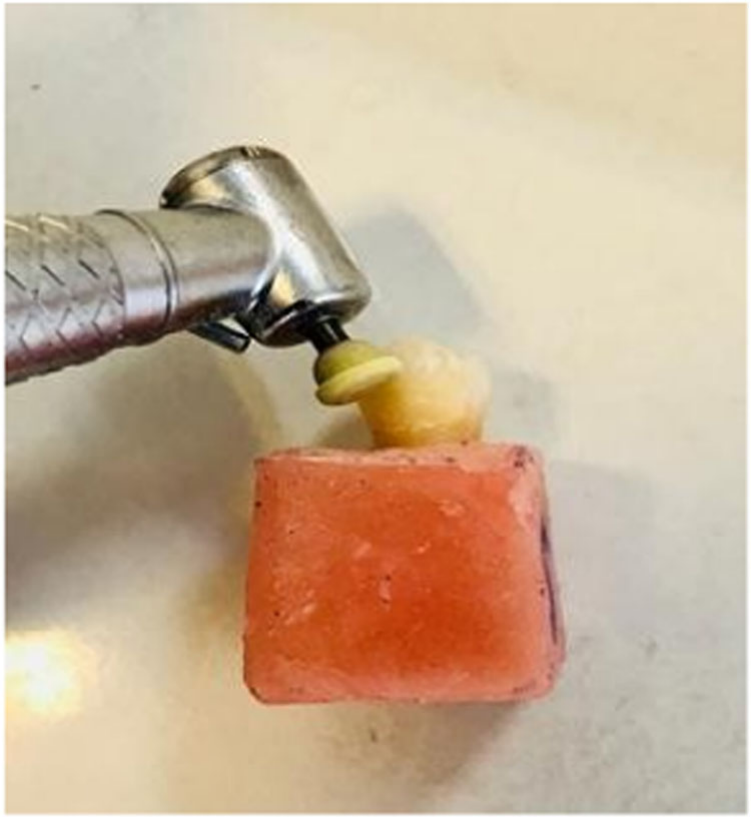
Polishing.

All the teeth were bonded with Adper single bond 2 (3M, ESPE). The adhesive was applied to the cavity of each tooth in two layers using a microbrush (Permium) and air‐dried to thin the bonding layer of the surface; Then, it was cured in 20 s. A nanohybrid composite (Z350 3M ESPE) was placed into the class V cavity and light cured in 40 s using the LED (Light curing unit; Demi plus) with the light intensity at 1200 $\mathrm{mW}/{\mathrm{cm}}^{2}$ throughout the study. The distance between the light curing unit tip and the composite was less than 1 mm. All the teeth were stored in distilled water in separate tanks for 24 h at 24°C (room temperature). Then, the surface of all the restorations was finished burs and fine polished carefully with polishing disks (Shofo) (Figure 3).

To stimulate the oral condition, a thermocycling machine (TC‐300; Vafaie Industrial) was used for 500 cycles at 5 ± 2/50 ± 2°C with a dwell time of 30 s. After that, the teeth were stored in distilled water in separate tanks. Then, the teeth surfaces were coated with nail varnish (two layers), except for 1 mm from the border of the restoration. During the application of nail varnish, a moist sponge was placed on it to protect the restoration from desiccation. Then, they were stored in 2% basic fuchsine tank solution (Merck) for 24 h at room temperature (24°C). The superficial dye of specimens was washed with running water.

**Figure 4:**
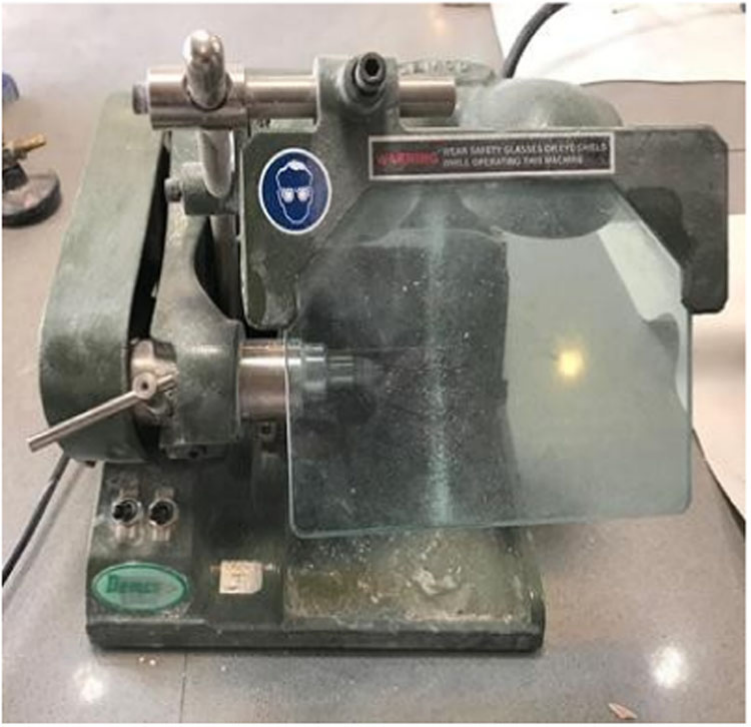
Nonstop cutting machine.

To remove acrylic cubes, we sectioned the teeth horizontally. Then, the teeth were cut longitudinally in a buccolingual direction from the middle line of the restorations using a diamond disk (Microdont in a nonstop cutting machine (Demco E96; CMP Industries) under a cold water spray (Figure 4).

**Figure 5:**
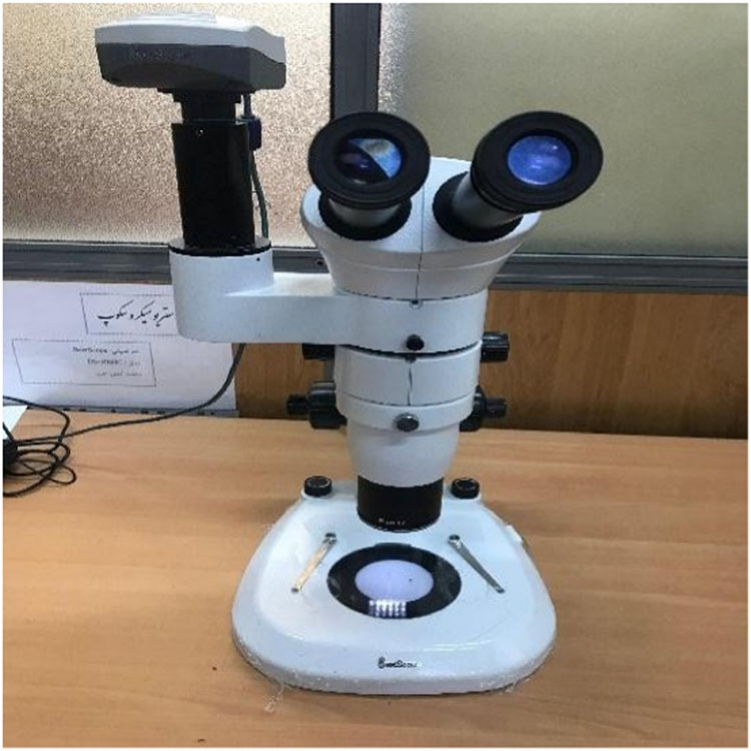
Stereomicroscope.

The sectioned surface was examined with a stereomicroscope (BS‐3060C; BestScope) at ×40 (Figure 5). Two blinded trained examiners who were both educated and talented dentistry students measured the amount of dye penetration at both occlusal and gingival margins using the microleakage scores (Sharafeddin et al., 2019).

0 = without dye penetration;

1 = penetration of dye up to one‐half of the distance between the restoration margin and the axial wall;

2 = penetration of dye extending beyond one‐half but not reaching the axial wall; and

**Figure 6:**
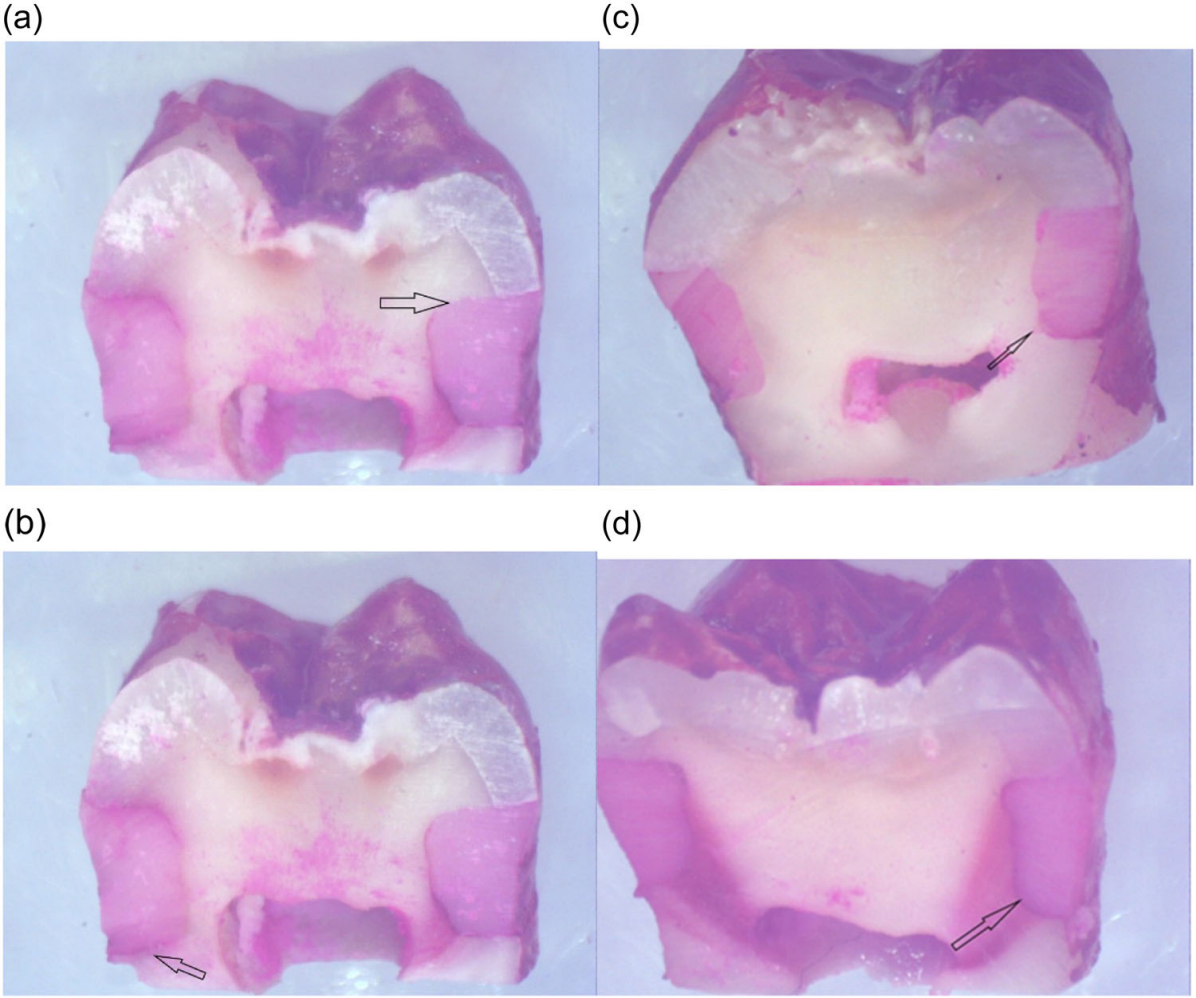
Displays the sectioned teeth (×40 magnification). (a) Score 0: Phosphoric acid 37% group. (b) score 1: Bromelain enzyme 10% group. (c) score 2: Papain enzyme 10% group. (d) score 3: CO_2_ laser group.

3 = penetration of dye reaching the axial wall (Figure 6).

Intraexaminer reliability was evaluated using STATA statistical software by computing weighted kappa. Kappa statistic showed acceptable intraexaminer reliability. The value of weighted kappa was 0.75.

For statistical analysis, Kruskal–Wallis, Mann–Whitney, and post hoc tests with Bonferroni correction were used to compare the microleakage value between groups. We used the chi‐squared test for qualitative evaluation (*p* < 0.05).

The power of statistical tests was more than 75%. The significance level was set at 0.05.

## 3 RESULTS

**Figure 7:**
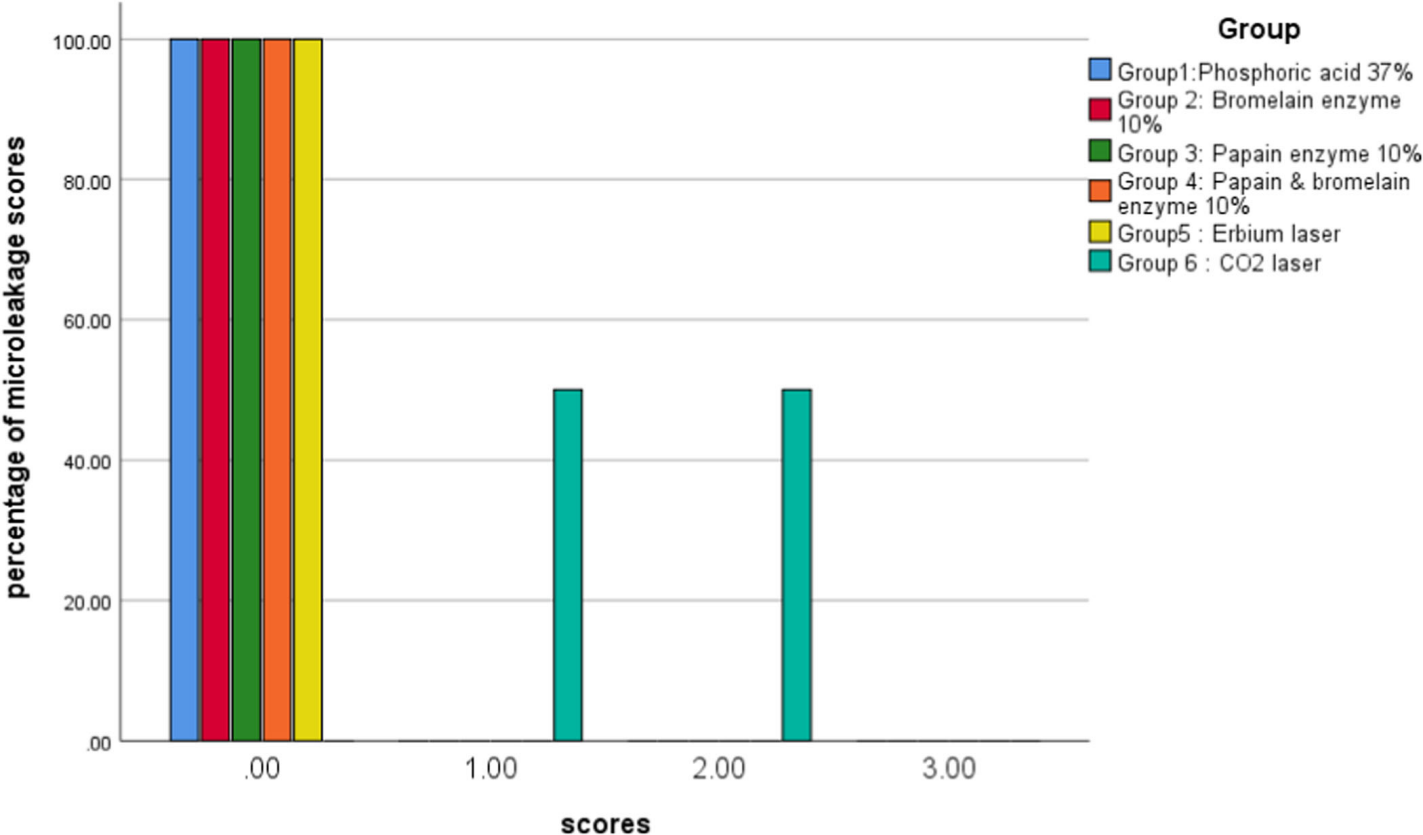
The scores and percentage of occlusal (dentin margin) microleakage in experimental groups. 0 = no dye penetration. 1 = dye penetration between the restoration and tooth up to one‐half of the distance between the tooth surface and the axial wall. 2 = dye penetration extending beyond one‐half but not reaching the axial wall. 3 = dye penetration reaching the axial.

**Figure 8:**
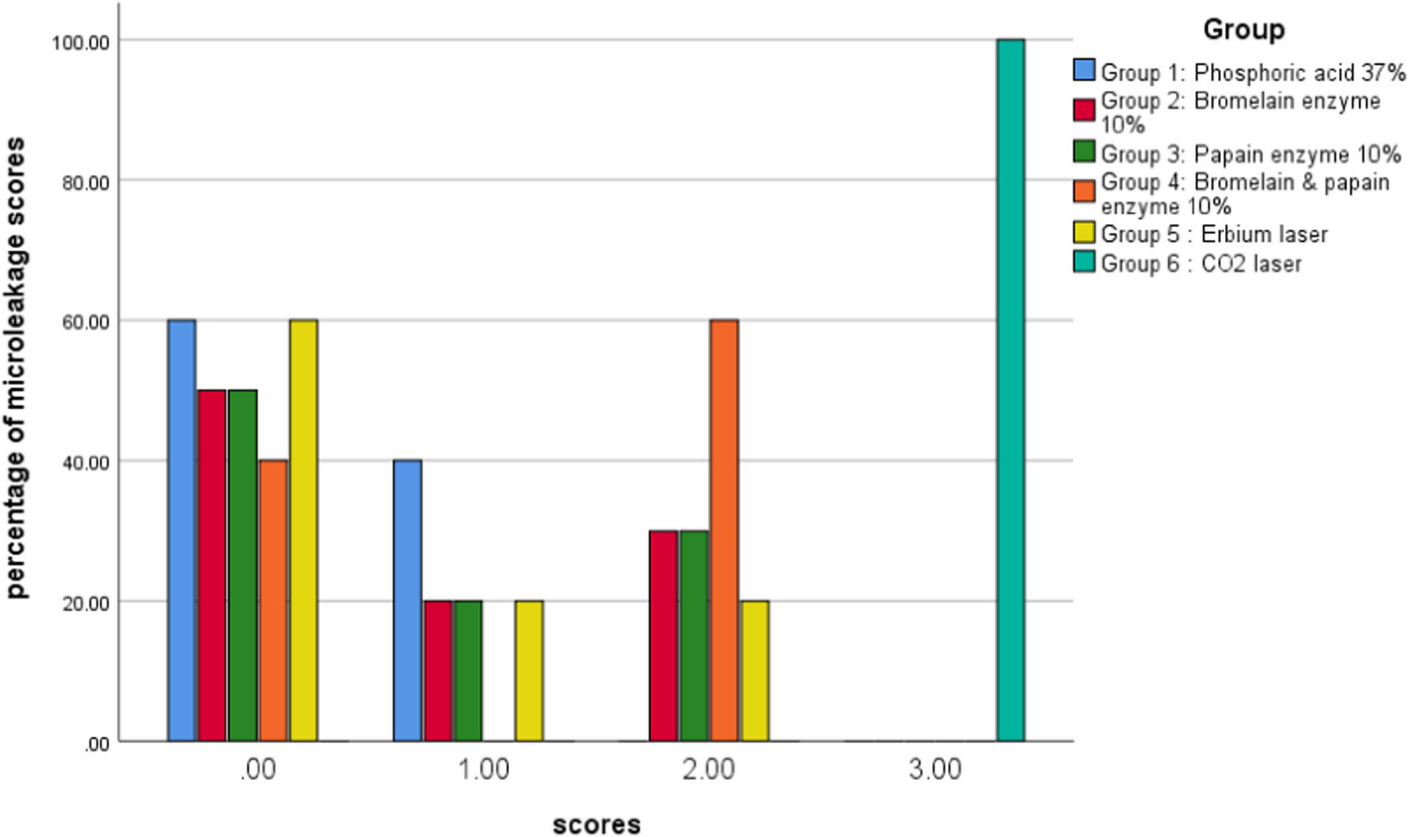
The scores and percentage of gingival (dentin margin) microleakage in experimental groups. 0 = no dye penetration. 1 = dye penetration between the restoration and tooth up to one‐half of the distance between the tooth surface and the axial wall. 2 = dye penetration extending beyond one‐half but not reaching the axial wall. 3 = dye penetration reaching the axial.

Results of the Mann–Whitney test showed that in occlusal margins CO_2_ laser had the most microleakage (mean = 1.50) and the other five groups had no microleakage (mean = 0.00). In gingival margins, phosphoric acid 37% had the lowest microleakage (mean = 0.40) and CO_2_ laser had the most microleakage than the other groups (mean = 3.00) and it was statistically significant (*p* < 0.001). Based on the Kruskal–Wallis test, in both margins, comparison of microleakage among groups 1, 2, 3, 4, and 5 showed no significant differences (*p* = 1) and group 6 revealed a significant difference with other groups (*p* ˂ 0.001). The results are presented in Tables 2, 3, 4, 5, also in Figures 7 and 8.

**Table 2:** Mean ± SD and minimum and maximum microleakage scores in occlusal margins.

Groups	Mean ± SD	Minimum	Maximum	*p* Value
**Group 1** Phosphoric acid 37%	0.00 ± 0.000	0	0	
**Group 2** Bromelain enzyme 10%	0.00 ± 0.000	0	0	
**Group 3** Papain enzyme 10%	0.00 ± 0.000	0	0	<0.001^b^
**Group 4** Bromelain and papain enzyme 10%	0.00 ± 0.000	0	0	
**Group 5** Erbium laser	0.00 ± 0.000	0	0	
**Group 6** a CO_2_ laser	1.50 ± 0.527	1	2	

*Note*: 0 = no dye penetration, 1 = dye penetration between the restoration and tooth up to one‐second of the distance between the tooth surface and the axial wall, 2 = dye penetration extending beyond one‐second of the distance but not reaching the axial wall, 3 = dye penetration reaching the axial wall in the occlusal margin.

a Group 6 (CO_2_ laser) had a significant difference with other groups. Mann–Whitney tests with Bonferroni correction test as post hoc test, *p* < 0.001.

^b^ Kruskal–Wallis test.

**Table 3:** Mean ± SD and minimum and maximum microleakage scores in gingival margins.

Groups	Mean ± SD	Minimum	Maximum	*p* Value
**Group 1** Phosphoric acid 37%	0.40 ± 0.516	0	1	
**Group 2** Bromelain enzyme 10%	0.80 ± 0.919	0	2	
**Group 3** Papain enzyme 10%	0.80 ± 0.919	0	2	<0.001^b^
**Group 4** Bromelain and papain enzyme 10%	1.20 ± 1.033	0	2	
**Group 5** Erbium laser	0.60 ± 0.843	0	2	
**Group 6** a CO_2_ laser	3.00 ± 0.000	3	3	

*Note*: 0 = no dye penetration, 1 = dye penetration between the restoration and tooth up to one‐second of the distance between the tooth surface and the axial wall, 2 = dye penetration extending beyond one‐second of the distance but not reaching the axial wall, 3 = dye penetration reaching the axial wall in the occlusal margin.

a Group 6 (CO_2_ laser) had a significant difference with other groups. Mann–Whitney tests with Bonferroni correction test as post hoc. *p* < 0.001.

^b^ Kruskal–Wallis test.

**Table 4:** The occlusal microleakage scores numbers and percent.

Scores	Materials							
	Phosphoric acid	Bromelain	Papain	Bromelain + Papain	Erbium laser	CO_2_ laser	Total (%)	*p* Value
0	100% 10	100% 10	100% 10	100% 10	100% 10	0% 0	83.33% 50	
1	0% 0	0% 0	0% 0	0% 0	0% 0	50% 5	8.33% 5	<0.001^a^
2	0% 0	0% 0	0% 0	0% 0	0% 0	50% 5	8.33% 5	
3	0% 0	0% 0	0% 0	0% 0	0% 0	0% 0	0% 0	
Total (*n*)	10	10	10	10	10	10	100 60	

^a^ Chi‐squared test.

**Table 5:** The gingival microleakage scores numbers and percent.

Scores	Materials							
	Phosphoric acid	Bromelain	Papain	Bromelain + Papain	Erbium laser	CO_2_ laser	Total (%)	*p* Value
0	60% 6	50% 5	50% 5	40% 4	60% 6	0% 0	43.33% 26	
**1**	40% 4	20% 2	20% 2	0% 0	20% 2	0% 0	16.66% 10	<0.001^a^
2	0% 0	30% 3	30% 3	60% 6	20% 2	0% 0	23.33% 14	
3	0% 0	0% 0	0% 0	0% 0	0% 0	100% 10	16.66% 10	
Total (*n*)	10	10	10	10	10	10	100 60	

^a^ Chi‐squared test.

## 4 DISCUSSION

Longevity of class V resin restorations depends on the quality of the bond at the cavosurface margin. The zone just under the cementum has fewer dentinal tubules and may have a low permeability to adhesive resins, even after conventional acid etching. Dentin conditioning with acidic material may demineralize more of the dentine surface, so the applied adhesive resin monomers can infiltrate and produce a weaker hybrid layer at this important interface (Andermatt & Özcan, 2021). With the purpose of eliminating the influence of the organic matrix on the adhesion of the composites to the dentin, various substances with deproteinizing characteristic were suggested, like papain and bromelain enzymes (Sharafeddin & Safari, 2019).

In the present study, no significant difference was observed in the microleakage between phosphoric acid 37% and those treated with bromelain enzyme 10%. Kasraei et al. (2017) indicated that there was no significant difference in microleakage between the bromelain‐treated and acid‐etched surfaces. Dayem and Tameesh (2013) revealed that using bromelain enzyme on acid‐etched dentin significantly decreased the microleakage because they used bromelain on acid‐etched dentin and we used bromelain on the dentin. These results may be explained by differences in the effect of other influential criteria such as duration and percentage of the use of bromelain. In our study, we used 10% only bromelain for 60 s on the dentin, and Dayem et al. used pure bromelain enzyme for one 60 s on the surface of the dentin. This result may be due to the ability of the bromelain enzyme to eliminate the collagen fiber from the acid‐etched dentin surface competently, and this will increase the diffusion efficiency of the resin monomer to the sound dentin and minimize the microleakage. The use of bromelain after acid etching decreased the microleakage in comparison to bromelain alone. This is because the bromelain enzyme induces the permeability of the dentin surface by reduction of collagen from the acid‐etched dentin surface and leads to the widening of the dentinal tubules in the outer surface and also increases the surface energy and infiltration and penetration of monomers. Since collagen has low and hydroxyapatite has high surface energy, the removal of collagen from the etched dentin reduces the organic material, increases the surface energy, and alternates the hydrophilic properties of the dentin, leading to more penetration of adhesive resin into the dentin (Chauhan et al., 2015).

In 2003, papain (Dawkins et al., 2003) was presented in dentistry. The product, Papacárie, is used in the removal of caries with chemical effect; infected tissues are eliminated very safely without causing any harm to the healthy structures in the oral cavity, using hand instruments without cutting edges instead of rotary instruments. Pithon et al. (2012) suggested that the use of 10% papain as a deproteinizing agent before acid etching increases the shear bond strength because of the removal of organic elements. Khattab and Omar (2012). used papacariѐ for caries removal before the etch and bond system and they indicated that papacarié improves the bond strength of the composite resin restorations. However, we can claim that increasing the bond strength could probably decrease the microleakage. In our study, papain, as the treatment agent, decreased the microleakage. The differences in our results and those of the previous studies can be related to their use of papain on the acid‐etched surface. We can conclude that the papain could improve micro‐morphological changes to the collagen network. Thus, the enhanced bond strength of the composite resin to the dentin surface could be attributed to the clearly distinguishable hybrid layer formed at the dentin resin interface after caries removal using Papacarié, which allowed the accomplishment of a durable bond (Lopez et al., 2007).

Recently, more technologies for preparing the dentin and enamel surfaces of tooth have become widespread, like laser irradiation. It has been suggested the use of lasers for the treatment of dentin and enamel surfaces is an alternative to the conventional acid‐etching technique. Since the approval of Er:YAG lasers for cavity preparation, caries removal, and conditioning of tooth surface, there have been many studies on the use of this method in combination with composite resins (Donmez et al., 2013; Mozaffari et al., 2016).

In this study, we used Er:YAG and CO_2_ laser for surface treatment. Both of the two lasers are highly popular in dentistry. Evidence shows that the smear layer is completely melted and evaporated following Er:YAG laser irradiation, resulting in the deposition of insoluble salts at the orifice of the open dentinal tubules, which leads to their obstruction. On the other hand, CO_2_ laser concentrates high levels of energy in a small area. Energy is converted to heat and results in burning, melting, or vaporing of the respective area in the dentin. Melting and re‐crystallizing of the dentin result in obstruction of the dentinal tubules, and dentin hypersensitivity may be relieved (Basir et al., 2016).

Chiniforush et al. (2014) and Esteves‐Oliveira et al. (2008) found no significant difference in microleakage at the enamel margins between the specimens treated with Er:YAG laser and controls. The structure of the enamel treated with phosphoric acid and Er:YAG laser was similar to that of the enamel etched with phosphoric acid only. Mozaffari et al. (2016) assumed that with the use of Er:YAG laser, microleakage in the dentin margins was significantly higher than the unprepared surface. Chinelatti et al. (2006) reported greater microleakage at the margins of enamel in the restorations following surface treatment with Er:YAG laser in comparison to the surfaces not treated. They found that laser had an adverse effect on the seal of the restoration margin and increased the microleakage.

The exact mechanism of the action of the laser revealed that laser irradiation destroys the enamel prisms (Juntavee et al., 2013). Also, erbium laser by eliminating the smear layer and causing irregularities in the cavity walls, internal angles, and margins before adhesive system compromises the bond of the restorative material to the tooth structure and subsequently has adverse effects on the microleakage. In our study, the microleakage in Er:YAG laser‐treated surfaces was greater than phosphoric acid‐etched surfaces, but the difference was not significant and microleakage in the margins of dentin was significantly higher than the enamel margins. The differences in results might be due to the fact that after laser irradiation, we did not use acid etch, but in Chinelatti's study, they used acid etch after erbium laser.

CO_2_ laser has been employed in clinical and laboratory studies for soft and hard tooth structures (Kasraei et al., 2018). On the hard dental structure, CO_2_ laser induces chemical and physical micro‐morphological changes, melting of the enamel prisms and re‐crystallization, with the formation of small bubble‐like inclusions and pores (Wahle and Wend) 1993). In Mozaffari et al.'s study (2016), the application of CO_2_ laser did not increase the microleakage at the dentin and enamel or margins. However, in our study, CO_2_ laser increased the microleakage, and the mean of microleakage scores in the CO_2_ laser group was significantly higher than other tested groups in the gingival and occlusal margins. The difference between their study and ours is that they used acid etch after laser and we only treated the surface with laser. When CO_2_ laser was irradiated, the possible alteration of the surface morphology, like melting and recrystallization, occurred and some dentinal tubules were obstructed (Basir et al., 2016). After laser irradiation, when the surface was acid etched, this obstruction did not interfere with bonding procedures, so the laser did not adversely affect the bonding procedures and did not increase the microleakage. This difference may be related to the potential of phosphoric acid etching in penetrating and removing the modified surface.

In our study, the amount of microleakage at the enamel margin was less than the dentin margin. The different levels of microleakage are because of the difference in the composition of the dentin and enamel; hence, lack of enamel at gingival margins results in more microleakage in comparison to the occlusal margins (Hottmann et al., 2002). A higher concentration of organic ingredients of the dentin and its tubular structure may interfere with the adhesion process (Eliades et al., 1997). Additionally, in the class V cavity, the dentinal tubules are arranged roughly parallel to the gingival margin; therefore, the classical structure of the hybrid layer is damaged, and the microleakage in gingival margins occurs more than in the occlusal margin (Wang & Spencer, 2002). According to the results of Kasraei et al. (Pithon et al., 2012). Dentin margins revealed significantly higher microleakage than the enamel margins, but there was no significant difference in the microleakage of enamel and dentin margins among the three groups. Eventually, they concluded that application of 5% of bromelain enzyme and Nd:YAG laser on phosphoric acid‐etched dentin before the application of adhesive has no significant effect on the marginal microleakage of class V composite resin restorations. Apel et al. (2005) showed that subablative erbium laser irradiation can result in the formation of altered organic and nonorganic matrixes such as melted collagen fibrils, calcium pyrophosphate, calcium metaphosphate, and alpha and beta tri‐calcium phosphate with variable degrees of acidic solubility. Therefore, lased dentin probably cannot be etched completely. As a result, the quality of micromechanical interlocking and penetrating of the bonding agent into the dentin decreased and microleakage in the gingival margins increased. Consequently, the mean of microleakage in only bromelain‐treated surfaces was higher than in phosphoric acid‐etched surfaces. If bromelain is applied on the acid‐etched surfaces, it could have a better effect on decreasing the microleakage because the bromelain enzyme, by removing collagen fiber from the acid‐etched substrate and widening the dentinal tubules (Omae et al., 2007), increases the permeability of the dentin surface in the superficial surface and increases the energy of the surface, causing better penetration of adhesive resin into the dentin and better bond.

In our results, the microleakage in the surfaces treated only with papain was higher than the phosphoric acid group because we did not use acid etch after papain. Although the application of papain before etching induces micro‐morphological changes to the collagen fibrils, eliminates the smear layer, and exposes dentinal tubules, it enhances the bond strength. The mean of microleakage in the surfaces treated with only erbium laser was higher than the phosphoric acid‐etched ones, so it could not decrease the microleakage only. Before using acid etching, erbium laser with the removal of the smear layer and production of tooth surface irregularities may be able to decrease the marginal microleakage (Costa Santos et al., 2019). However, in our study, we did not use acid etch after erbium irradiation. The differences in laser criteria can significantly influence the results and influence the marginal microleakage of the composite restorations as well. Marotti et al. (2010). showed that surface pretreatment with only 1 W laser decreased the microleakage; in our study, we used 1.5 W erbium laser. CO_2_ laser irradiation without acid etching significantly increased the microleakage in both margins. In Mozaffari et al.'s study (Mozaffari et al., 2016), the use of CO_2_ laser before acid etching had no adverse effect on the seal of the margin. Because the laser altered the surface morphology with melting, recrystallization, and closure of some tubules, the capability of acid etching in removing and penetrating the alternated surface increased (Meshram et al., 2019; Sharafeddin & Maroufi, 2022). And better acid etching may lead to a better bond and decrease the microleakage. Sharafeddin et al. evaluated Er:YAG laser and CO_2_ laser irradiation and bromelain and papain 10% enzyme in composite resin restorations shear bond strength (SBS) and compared it with acid‐etched cavities in human molar teeth. They reported that the use of CO_2_ laser on the dentin surface in composite restoration did not enhance the SBS and had a negative effect on it and decreased the SBS due to its thermal damage to dentin (Meshram et al., 2019). CO_2_ laser has some different thermal effect that is sometimes caused, making this device inappropriate for improving tooth‐colored restoration (Laky et al., 2023; Marracini et al., 2005). It has been reported that the use of CO_2_ laser not only increases the SBS but can also have thermal adverse effects. In general, in laser treatment, different factors might affect the tooth structure, such as the nature of the laser, exposure time, laser power, pulse duration, wavelength, amount of air steam created by water, and the distance of the laser tip from the tooth structure (Shinoki et al., 2011). This factor also may affect enamel and dentin microleakage (Laky et al., 2023). For future studies, we suggest further in vivo studies using different types of bonding systems, different concentrations of enzymes, and different laser setting parameters in enamel and dentin.

## 5 CONCLUSION

According to the current study, it can be concluded that microleakage scores of phosphoric acid 37% were significantly lower than the other groups. Although papain and bromelain 10% enzyme had an effect on removing the collagen matrix, it did not decrease the microleakage in comparison to phosphoric acid 37%. The use of CO_2_ laser alone for surface treatment of composite resin increased the microleakage. Erbium laser had a positive effect on the microleakage decrease in occlusal and gingival margins.

## AUTHOR CONTRIBUTIONS

Farahnaz Sharafeddin conceptualized the study. Anahita Fadaei Tabrizi and Farahnaz Sharafeddin prepared the specimens and did the data collection, and both of them wrote the original draft. Farahnaz Sharafeddin revised the first editing of the manuscript. Farahnaz Sharafeddin supervised the study. All authors have read and agreed to the submitted version of the manuscript.

## CONFLICT OF INTEREST STATEMENT

The authors declare no conflict of interest.

## Data Availability

The data used to support the findings of this study are included within the article.
